# Classification and treatment strategy for Moyamoya disease-related aneurysms

**DOI:** 10.1186/s41016-023-00352-1

**Published:** 2023-12-20

**Authors:** Yangchun Hu, Xiaojian Wang, Chao Li, Liang Zhao, Jing Luo, Lei Ye, Baochun Cheng

**Affiliations:** Department of Neurosurgery, The First Affiliated Hospital of Anhui Medical University, Jixi 218, Hefei, 230022 People’s Republic of China

**Keywords:** Moyamoya disease, Aneurysm, Clipping, Revascularization, Endovascular embolism

## Abstract

**Background:**

Moyamoya disease (MMD) is a cerebrovascular disorder characterized by progressive unilateral or bilateral stenosis of the distal internal carotid artery. As hemodynamic features in MMD patients alter, the comorbidity of intracranial aneurysm (IA) is sometimes observed clinically. We aim to investigate clinical characteristics and therapeutic strategies for the comorbidity of Moyamoya disease with intracranial aneurysms (MMD-IA).

**Methods:**

A total of 13 MMD-IA patients were recruited in this study and were manifested to be intracranial hemorrhage. We reviewed the surgical technique notes for all patients.

**Results:**

According to the locations of an aneurysm, MMD-IA could be divided into several categories: (1) MMD-IA at a circle of Willis—aneurysms usually located at the trunk of Willis circle; (2) MMD-IA at collateral anastomosis—aneurysms located at the distal end of collateral anastomosis; and (3) MMA-IA at basal ganglia region. In this report, aneurysms in 10 patients located at Willis circle, 2 at the pericallosal artery, and 1 at the basal ganglia region. Among them, endovascular embolism was performed among 5 patients. Aneurysm clipping was conducted among 7 patients. A patient with an aneurysm at the basal ganglia region just accepted revascularization treatment. All the treatments were successful. Follow-up studies, ranging from 6 to 24 months, demonstrated all patients received satisfactory curative effects.

**Conclusion:**

Diverse clinical presentations could be observed among MMD-IA patients. Individualized neurosurgical treatments should be chosen according to the locations of the aneurysm.

## Background

Moyamoya disease (MMD) is a cerebrovascular disorder characterized by progressive unilateral or bilateral stenosis of the distal internal carotid artery (ICA), usually extending to the anterior cerebral arteries (ACA) and middle cerebral arteries (MCA) with unclear etiology [1–3]. The incidence of MMD varies throughout districts, with the highest rate in East Asia, such as Korea, Japan, and China [4]. Epidemiologically, the onset age of MMD presents a bimodal distribution among general populations. A large peak of onset centers is around 5 years old and a relatively small peak at 40 years old [5]. Although both child and adult MMD patients would cause ischemic syndromes, it has been reported that repeated transient ischemic attacks (TIA) or strokes were much more commonly found in child patients, while adult patients often presented intracranial hemorrhage [6]. The most common types of hemorrhagic MMD are intraventricular hemorrhage (IVH) and intracerebral hemorrhage (ICH), which are attributed to periventricular fragile collateral vessels [7]. Furthermore, the formation of MMD-related intracranial aneurysms (IA) occurs frequently and is considered a consequence of increased hemodynamic stress through relatively narrow caliber collateral networks [8, 9]. However, due to the complex pathogenic mechanisms of MMD, there have been just several hypotheses explaining the pathogenesis, making its prevention and medication intractable.

The comorbidity prevalence of MMA with IA (MMA-IA) is quoted between 3.4–14.8% [10–12]. Pathophysiology of the comorbidity is complex and is speculated to be correlated with a combination of compensatory hemodynamic stress, abnormal vascular wall architecture, and anatomic location. The formation of IA and unstable blood flow are thought to be the predominant factors of hemorrhagic MMD [8, 10, 12]. However, the rate of IA-relating ruptures varies between studies. Rhim et al. reported that the rupture rate of MMD-related IA was 24.7% (19/77) [13]. However, Kim et al. found the rate could be as high as 63.6% (7/11) in the same population [7]. Furthermore, Kawaguchi found the rupture rate could be as high as 89% (99/111) in the Japanese population [11]. We think the diverse results among studies may be attributed to different IA locations.

MMD-related IA occurs almost in all cerebrovascular distributed intracranial areas, ranging from the circle of Willis to Moyamoya vessels [14]. As different pathogenesis and hemodynamic features are found among IA of different locations, MMD-IA is usually categorized by the location which could facilitate the decision of treatment strategies [15]. Two main subtypes of MMD-related IA have been determined: IA in major arteries and IA in peripheral arteries [16]. Several therapeutic options can be currently decided according to the aneurysm locations, including direct clipping, revascularization, and endovascular embolism. However, each therapeutic option is reported by case report alone, and there is still no consensus for therapeutic strategies for the comorbidity of MMD with IA.

In this study, we retrospectively report the management of MMD-related IA and focus on the experiences of treatment strategies according to the locations of the IA.

## Methods

### Patients

**Fig. 1:**
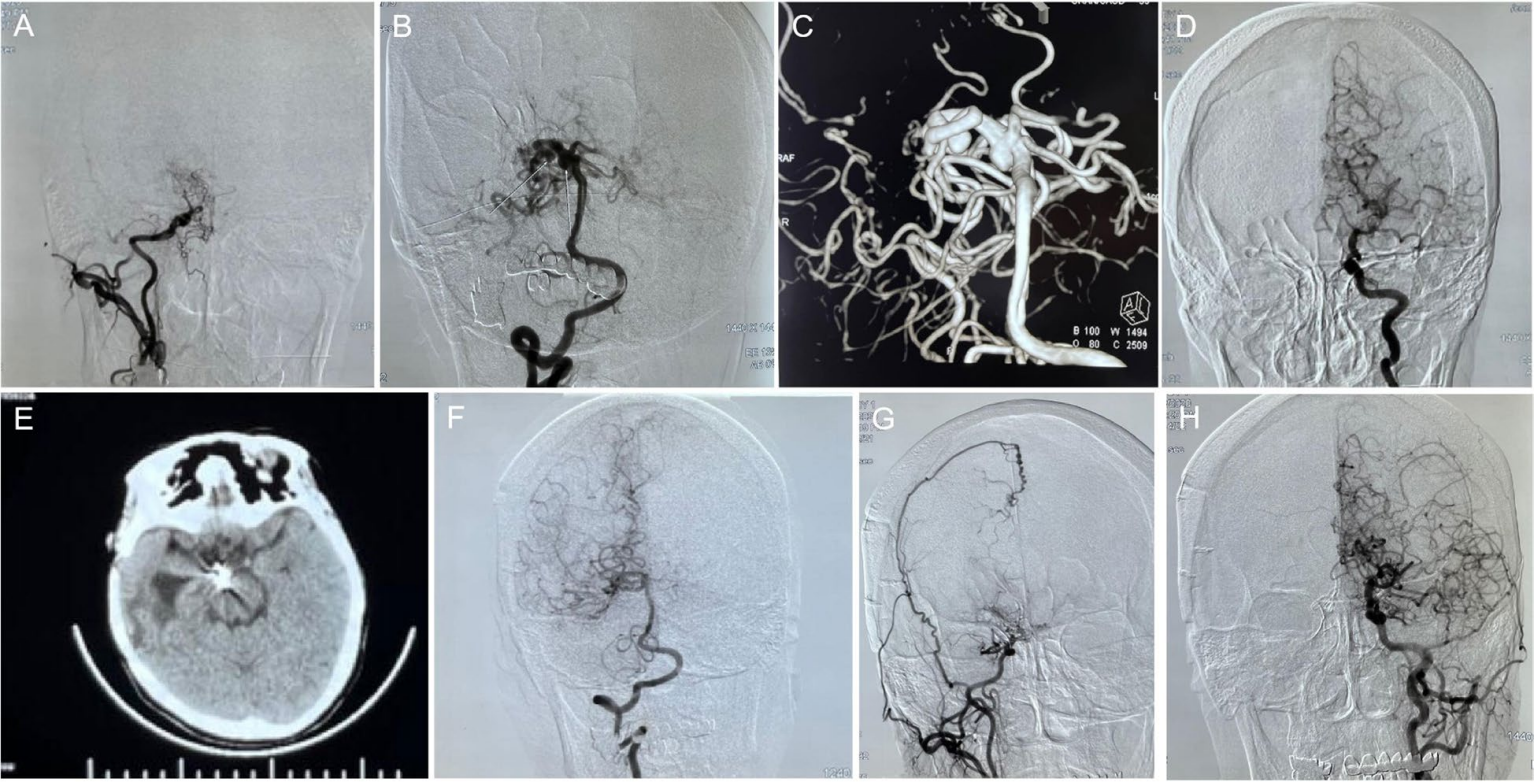
Typical magnetic resonance imaging for Moyamoya disease (MMD)-related intracranial aneurysm (IA). **A**–**D** figures referred to the preoperative neuro-image, and **E**–**H** figures referred to the postoperative neuroimage. **A** Digital subtraction angiography (DSA) test for the right common carotid artery (CCA) showed the MMD lesion. **B** DSA test for the right vertebral artery (VA) showed 2 aneurysms. **C** 3D-DSA for the VA. **D** DSA for the left internal carotid artery (ICA). **E** Postoperative computer tomography (CT) scan. **F** Postoperative DSA test for the right (VA). **G** Postoperative DSA test for the right CCA. **H** Postoperative DSA test for the left ICA

A total of 82 MMD patients were recruited and treated in our department between August 2015 and August 2019. All patients were diagnosed with MMD by the neuro-imaging results according to the Ministry of Health and Welfare in Japan. Among all MMD patients, 13 patients were simultaneously verified with IA, with an incidence of 15.9% (13/82), including 6 males (age ranging from 49 to 62 and mean age of 53.0 ± 6.2) and 7 females(age ranging from 42 to 64 and mean age of 50.6 ± 7.5). Among them, the clinical manifestation of 5 patients exhibited light coma, with a GCS of 11.5 ± 3.1, while the other 8 patients exhibited headache, drowsiness, and signs of meningeal irritation, with a GCS of 13.9 ± 2.9. Computed tomography (CT) scans in other hospitals indicated the intracerebral hemorrhagic signs, which consisted of 10 subarachnoid hemorrhages (SAH) and 3 intraventricular hemorrhages (IVH). Whole brain digital subtraction angiography (DSA) revealed various degrees of steno-occlusion signs in bilateral or unilateral internal carotid (ICA), anterior cerebral artery (ACA), or middle cerebral artery (MCA). Collateral circulations were extensively observed intracranially, accompanied by IA. Among the 13 patients, 5 patients had multiple aneurysms. Meanwhile, 10 aneurysms are located in the circle of Willis, 2 aneurysms are located in the pericallosal artery, and 1 aneurysm is located in the basal ganglia region. Typical neuro-imaging data for MMD-related IA was depicted in Fig. 1. Detailed demographic and clinical characterizations of each patient were summarized in Table 1. Informed consent was obtained from all individual participants included in the study. This study was approved by the Human Research Ethics Committee and conducted within the confines of the 1964 Helsinki Declaration and its later amendments or comparable ethical standards.

**Table 1:** Summary of clinical features of 13 patients of MMD-associated IA

NO.	Gender	Age	Clinical manifestation at admission	Hunt-Hess score	Numbers of IA	Location	Treatment strategy
1	Male	49	Bleeding	II	Multiple	Left PCA and right ICA	Clipping and revascularization
2	Male	49	Bleeding	I	Mono	Right MCA	Endovascular embolism and revascularization
3	Female	64	Bleeding	III	Multiple	Left ACOA and left PCOA	Clipping and revascularization
4	Female	52	Bleeding	II	Multiple	Right ACOA	Clipping and revascularization
5	Female	46	Bleeding	III	Multiple	Right PCA	Clipping and revascularization
6	Female	56	Bleeding	III	Mono	Left PCA	Endovascular embolism and revascularization
7	Male	62	Bleeding	II	Mono	Left MCA	Clipping and revascularization
8	Female	42	Bleeding	II	Multiple	Left PCOA	Endovascular embolism and revascularization
9	Female	46	Bleeding	I	Mono	Left PCA (Basal ganglia region)	Revascularization
10	Male	52	Bleeding	III	Mono	Left ACOA	Endovascular embolism and revascularization
11	Female	48	Bleeding	III	Mono	Left ACA	Endovascular embolism and revascularization
12	Male	46	Bleeding	II	Mono	Left MCA	Clipping and revascularization
13	Male	52	Bleeding	II	Mono	Left PCOA	Clipping and revascularization

### Characterizations of MMD-associated IA and definition of treatment outcomes

The characterizations of MMD-associated IA were recorded in Table 2, including aneurysmal sizes, types of aneurysmal neck, and morphologies. In this study, we performed a DSA test for all patients who received operation treatment at 3 months after the operation. We defined the efficacy of treatment in that the neuro-imaging had no signs of relapse and rebleeding, and meanwhile, the patients were self-reported to be free of symptoms.

**Table 2:** Characteristics of MMD-associated IA

Characteristics	Total	Anterior circulation IA	Post circulation IA
No. of lesions			
Size			
< 3 mm	11	8	3
3–5 mm	3	2	1
Aneurysmal neck			
Broad	11	7	4
Narrow	3	3	0
Morphology			
Saccular	5	4	1
Fusiform	9	5	3
Irregular	1	1	0

## Results

### Surgical strategies

In the treatment strategies for the patient, endovascular embolism with subsequent revascularization was displayed among 5 patients by the senior neurosurgeons. Aneurysm clipping with subsequent revascularization was conducted among 7 patients. The patient with an aneurysm in the basal ganglia region just underwent revascularization, while the aneurysm was unsolved. Additionally, 3 patients with IVH underwent external ventricular drainage treatment (Table 3).

**Table 3:** Treatment of modality for MMD-associated IA

Treatment options	Anterior circulation IA			Post-circulation IA		
	EVT+Rev	Clipping+Rev	Rev A	EVT+Rev	Clipping	Rev
Initial results for IA	4	6		3	1	
Complete occlusion	3	4	1	3	0	0
Near complete occlusion	1	1	0	0	1	0
Incomplete occlusion	0	1	0	0	0	0

*EVT+Rev* Endovascular embolism + revascularization, *Rev A* Revascularization alone

### Clinical outcomes and follow-up screening

We did follow-up interviews for all patients ranging from 6 months (short term) to 24 months (long term). All patients presented well excellent efficacy for imageological results. Three patients felt headaches at a short-term follow-up, but the symptoms vanished at a long-term follow-up. Furthermore, four patients felt memory deterioration at a short-term follow-up. Among them, two patients still felt memory deterioration at a long-term follow-up. Rebleeding did not occur in patients (Table 4).

**Table 4:** The complications of 13 patients in post-surgery and follow-up interview

Complication type	Cases of complication	
	Short-term	Long-term
Headache	3	0
Memory deterioration	4	2
Rebleeding	0	0

## Discussion

MMD is a progressive and occlusive cerebrovascular disorder of inconclusive etiology and is correlated with neither atherosclerosis nor inflammations [2, 17]. MMD is characterized with steno-occlusion at the terminals of bilateral or unilateral internal carotid arteries (ICAs), and/or the terminal branches, such as MCA and ACA, and meanwhile formation of collateral capillary networks [18]. Clinical manifestation varies among individuals of different ages. Generally, child patients usually present ischemic syndrome due to hypoperfusion, leading to repeated transient ischemic attack or stroke, while adult patients exhibit hemorrhagic symptoms [4, 19]. Headache is a most common but non-specific clinical presentation among MMD patients. Furthermore, MMD-related severity depends on the lesion locations [14]. Involvement in anterior circulation may lead to hemiparesis, speech dysfunction, and hemisensory impairments, while involvement in posterior circulations is more frequent and have greater disease severity, such as cognitive dysfunction, expressing fluency, and executive function [20, 21]. Among all complications in MMD, intracranial hemorrhage, which results from either fragile basal Moyamoya vessels or ruptured aneurysms, is the most life-threatening [7]. Previous reports have demonstrated that IA formation in MMD patients would be ranging from 3.4 to 14.8%, of which the incidence was significantly higher than the IA formation in normal patients. Progressive occlusion of ICA jeopardizes hemodynamic stress of its terminal and branch arteries, resulting in compensatory dilation and increases in blood flow through collateral vessels [22]. Therefore, IA formation would be considered as a consequence of stressed blood flow through relative narrow caliber collaterals. Meanwhile, due to the unstable hemodynamic stress in MMD, the patients undergo high bleeding risks. So, early treatment for both MMD and IA, aiming at improving hemodynamic stress, is the most urgent approach for reducing the risk of permanent neurological and cognitive deficits and improving a long-term prognosis and life expectancy, especially for patients who are symptomatic.

Despite the relatively high incidence of MMD-associated IA, therapeutic strategies are just found from a handful of case reports, and therefore, there was a lack of consensus on the treatment option, except for the surgical intervention. In clinical practice, the treatment strategy for MMD-associated IA depends on the location of IA. The difficulty is to assess the choice of treatment modality for each distinct disease process. Some investigations categorized MMD-associated IA into several groups based on the originality and location of IA, thus providing clues for different surgical options for MMD-associated IA [10, 15, 23]. The most accepted standard of classification helps the decision. (1) IA originating from major arteries, especially in the circle of Willis—in this kind of MMD-associated IA, IA is usually located in the posterior circulation, especially in the basilar artery. The condition is probably due to the increased stress of hemodynamics at posterior circulation and as a result of progressive occlusion at anterior circulation. (2) IA arising from Moyamoya vessels, namely peripheral IA—this kind of IA is prone to rupture and manifested with intraparenchymal hemorrhage or IVH [24, 25]. The major artery IA has no unified treatment to date. Surgical intervention is favorable, but some posterior arteries, such as wide neck BA and tip artery, are difficult to access. Additionally, it is hard to clip peripheral IA due to the difficulties in location. Endovascular therapy is sometimes a challenging option because of the tortuous approach of the parent artery. Revascularization is a potential approach, but needs to be further investigated.

In our study, we reported 13 MMD-associated IA patients among all 82 recruited MMD patients. The incidence of MMD-associated IA is 15.9% (13/82), with a gender ratio of 3:2 (male/female). Among them, two aneurysms are located in the pericallosal artery which was formed with the forward compensatory artery of the posterior circulation. Ten aneurysms are located in the circle of Willis, including 8 aneurysms in the posterior circulation and 3 aneurysms in the anterior circulation. An aneurysm is located in the basal ganglia region. The general constitution of MMD-associated IA was different from other case-cohort reports. Some reports demonstrated that MMD-associated IA had a high incidence in the posterior circulation of major artery IA [26]. We thought it might be due to the timing of DSA and different mechanisms of IA formation. Meanwhile, the ruptured aneurysms in main trunk arteries usually induced subarachnoid hemorrhage (SAH), while ruptured aneurysms in peripheral arteries might lead to intraparenchymal hemorrhage. We noticed that the common location of aneurysms in MMD patients is still controversial. Larson et al. [27] reported 10 MMD-associated IA patients in a Caucasian cohort, and they found that 7 out of 10 aneurysms in the circle of Willis are located in the anterior circulation. However, in a meta-analysis concerning the MMD-associated IA, we found the posterior circulation was still the most common location for the aneurysms in MMD patients [28].

In the treatment strategies, 7/13 MMD-associated IA patients accepted direct microsurgical clipping, followed by revascularization treatment, and 5/12 patients underwent endovascular embolism, followed by revascularization treatment. The patient with the aneurysm located in the basal ganglia region just underwent a conservative revascularization treatment. All patients had good outcomes. Some previous investigations have summarized and suggested the treatment options for MMD-associated IA with different locations. Adams et al. [29] summarized some case reports and indicated that in the ruptured IA in anterior circulations, it was essential to perform the clipping or embolization treatment to prevent the re-rupture of IA. A meta-analysis study has summarized the treatment options for aneurysms in MMD patients [28]. For the IA located in the circle of Willis, most patients received open surgery (39.2%) and endovascular therapy (37.4%), in comparison with conservative therapy (23.4%). However, conservative therapy might be the most choice in the peripheral IA (43.1%) in comparison with endovascular therapy (26.5%) and open surgery (30.4%). These results were similar to our study.

Meanwhile, collateral networks should be carefully preserved to ensure sufficient cerebral perfusion. In another aspect, it remained controversial for unruptured aneurysms whether invasive treatments should be performed [30]. Some investigations have found that these unruptured IA might spontaneously disappear after the progressive steno-occlusion of the distal end of the ICA and the origin of the ACA or MCA, as MMD progressed [31, 32].

In another aspect, for the IA located in the posterior circulation, some clinical investigations tended to choose endovascular embolization treatment. However, although advanced techniques have been widely used recently, such as detachable coils and balloon- and stent-assisted techniques, it also had potential challenges for neurosurgeons [33, 34]. The endovascular embolization treatments for aneurysms in the superior cerebellar artery (SCA), P1-P2 junction of the posterior cerebral artery (PCA), or vertebrobasilar junction showed outstanding outcomes. However, the efficacy of using endovascular embolization treatment in basilar tip aneurysms was relatively worse [8].

Additionally, treatment options for the peripheral IA of AChA also included embolization with coils or glues and craniotomy. Clinically, neurosurgeons suggest to preserve the parent artery, when the aneurysm is located in the temporal horn of the lateral ventricle, to achieve satisfactory outcomes. However, the parent artery can be sacrificed when the aneurysm is located in the trigone of the lateral ventricle [35]. For the PChA aneurysm-associated MMD, craniotomy and endovascular embolization are effective [8]. We also noticed that the aneurysms, which cannot be accessed by direct surgical resection and endovascular embolization, could be treated with surgical revascularization [8].

In our study, we reclassified MMD-associated IA as (1) IA in/near the circle of Willis—because the aneurysm is located within the ICA system, surrounding the circle of Willis, direct clipping, followed by revascularization is a favorable choice. If the aneurysms are located in the vertebrobasilar artery system, it is difficult to be directly clipped. Therefore, endovascular embolism could be considered. (2) IA in the basal ganglia region—aneurysm in this region had a relatively minor risk of rupture, so preservation medication might be considered. (3) IA in the collateral anastomosis—aneurysms in this group are usually located within the brain tissues. It had difficulties with direct clipping. As the progression of collateral networks might lead to the disappearance of this kind of aneurysm with unknown reasons, revascularization alone for treatment of MMD or intra-aneurysmal endovascular embolism with subsequent revascularization treatment would be considered.

Additionally, in comparison with IA independently occurring in healthy individuals, arteries within the scope of neurosurgery among the MMD patients would be disorganized. Furthermore, MMD patients had decreased endurance of ischemia for brain tissue. Distraction of brain tissues during operations should be carefully avoided.

## Conclusions

In summary, diverse clinical presentations could be observed among MMD-IA patients. Individualized neurosurgical treatments should be chosen according to the locations of aneurysms.

## Abbreviations

ACA: Anterior cerebral arteries
CT: Computed tomography
DSA: Digital subtraction angiography
IA: Intracranial aneurysm
ICA: Internal carotid artery
ICH: Intracerebral hemorrhage
IVH: Intraventricular hemorrhage
MCA: Middle cerebral arteries
MMD: Moyamoya disease
PCA: Posterior cerebral artery
TIA: Transient ischemic attack
SAH: Subarachnoid hemorrhage
SCA: Superior cerebellar artery
